# Therapeutic potential of curcumin-integrated starch biomaterials in wound regeneration

**DOI:** 10.1007/s10856-026-07082-7

**Published:** 2026-05-26

**Authors:** Tilottoma Kargupta, Pooja N, Shreya Shahapur, Bhisham Narayan Singh, Nirmal Mazumder

**Affiliations:** 1 Department of Biophysics, Manipal School of Life Sciences, Manipal Academy of Higher Education, Manipal, India; 2 Department of Biotechnology, Manipal School of Life Sciences, Manipal Academy of Higher Education, Manipal, India

## Abstract

Bioplastics are an eco-friendly alternative to conventional plastics, with potential applications in the biomedical field. Curcumin, a bioactive compound present in *Curcuma longa*, has antioxidant, anti-inflammatory, and wound healing properties. In this study, potato starch/curcumin biopolymer films were synthesized and evaluated for their mechanical, functional, and biodegradation properties, followed by in vivo wound healing assessment in Wistar albino rats. The incorporation of curcumin increased the film thickness from 0.12 ± 0.01 mm to 0.16 ± 0.02 mm, the tensile strength from 3.25 ± 0.15 MPa to 4.18 ± 0.12 MPa, and the water contact angle from 15.31° ± 0.5° to 68.42° ± 0.8°, indicating enhanced hydrophobicity and mechanical stability. The water solubility decreased from 38.5 ± 1.3% to 24.6 ± 1.1%, whereas the moisture content decreased from 12.3 ± 0.6% to 7.8 ± 0.4%. In vivo studies of excision wounds (15 mm diameter) revealed that, by day 14, curcumin-incorporated films achieved 96.8 ± 1.2% wound closure compared with 82.5 ± 2.1% in untreated controls and 88.4 ± 1.5% with starch-only films. The wound healing performance was comparable to that of the marketed placental extract gel, with no signs of infection. These findings demonstrate that the integration of curcumin significantly improves the mechanical, barrier, and therapeutic properties of starch-based biopolymers, making them promising, cost-effective, and biodegradable wound dressing materials.

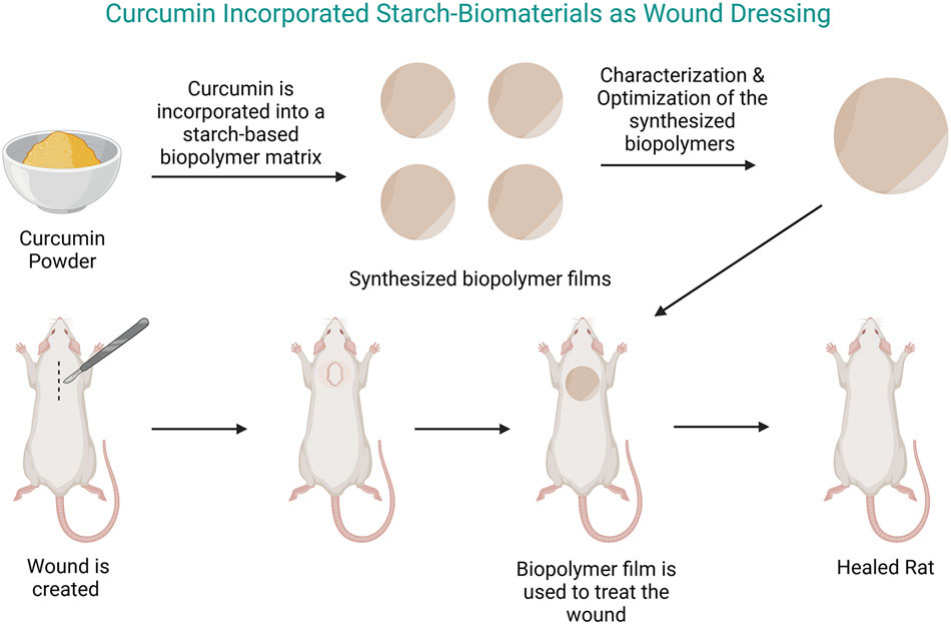

## Introduction

Plastics are mainly synthetic or semisynthetic organic polymers. They are lightweight, affordable, and have various applications, making them almost indispensable to the modern world [1]. Nonetheless, their toxicity to humans and the environment has led to a need for nontoxic, easily biodegradable, and more eco-friendly alternatives, which are preferable to conventional plastics [2]. Bioplastics are biologically derived polymer formulations that have properties similar to those of conventional petroleum-based plastics. Bioplastics are either biodegradable, based on organic monomers, or both. Several renewable resources could be utilized to produce bioplastics. Compared with conventional petroleum-based synthetic plastics, they are less toxic, have a lower carbon footprint, and have greater energy consumption efficiency. Materials made from microbial sources; inorganic materials such as carbon dioxide; and organic matter such as methane, cellulose, chitin, and starch can be used to synthesize bioplastics [3, 4].

Scientists and clinicians are trying to incorporate bioplastics into the medical field, with various reports of the efficiency of dextran and cellulose/chitosan-based polymers having worked successfully in wound healing [5]. *Curcuma longa*, colloquially known as turmeric, a perennial plant belonging to the Zingiberaceae family, is an important spice in Indian kitchens, with the first known mention as long as 4000 years during Vedic times [6, 7]. Curcumin, a yellow-colored polyphenol, is the bioactive component found in the turmeric rhizome. Curcumin has been found to have anti-inflammatory, antidiabetic, antiproliferative, anticancer, antithrombotic, and antirheumatic effects. There have been many studies on the use of curcumin to heal wounds because of its anti-inflammatory and antimicrobial properties. Curcumin also promotes collagen deposition and the proliferation of fibroblasts and helps scavenge reactive oxygen species that cause inflammatory effects [6, 8, 9]. Wound healing is a multifaceted process involving many distinct types of cells in the body. It is a dynamic process, and dysfunction in wound healing can cause infections and lead to necrosis of tissues, gangrene, and dermatitis. The removal of dead tissue and the provision of a sterile and moist environment are highly important when healing a wound. The transdermal application of medication has shown great promise in recent times to combat the low permeability of drugs across the skin in cases of conventional wound dressing treatments. Transdermal patches made of bioplastics have been reported to help close wounds efficiently with minimal scar tissue formation while keeping wounds free from microorganisms and moisture loss [10, 11].

Although several studies have explored starch-based biopolymers and the therapeutic potential of curcumin independently, there remains a lack of comprehensive studies integrating curcumin into starch matrices for simultaneous enhancement of mechanical properties and wound healing efficacy. In particular, limited work has systematically evaluated the combined physicochemical, functional, and in vivo wound healing performance of such systems.

Therefore, in this study, we aimed to develop and characterize curcumin-incorporated starch-based biopolymer dressings and evaluate their mechanical, functional, and biodegradation properties, along with their in vivo wound healing efficacy using a Wistar rat excision model.

## Experimental section

### Materials

Potato starch pure, curcumin (Turmeric yellow), and 2,2-diphenyl-1-picrylhydrazyl (DPPH) extra pure (95%) were obtained from Sisco Research Laboratories PVT. Ltd., India. 3-[4,5-Dimethylthiazol-2-yl]-2,5 diphenyl tetrazolium bromide (MTT) and glycerol were obtained from HiMedia Pvt. In addition, medical tape, self-adhesive bandages (3MTM CobanTM), Neosporin®, and Placentrex® gel were obtained from a local pharmacy in Manipal, Karnataka.

### Methods

#### Starch/curcumin dressing material preparation

**Fig. 1:**
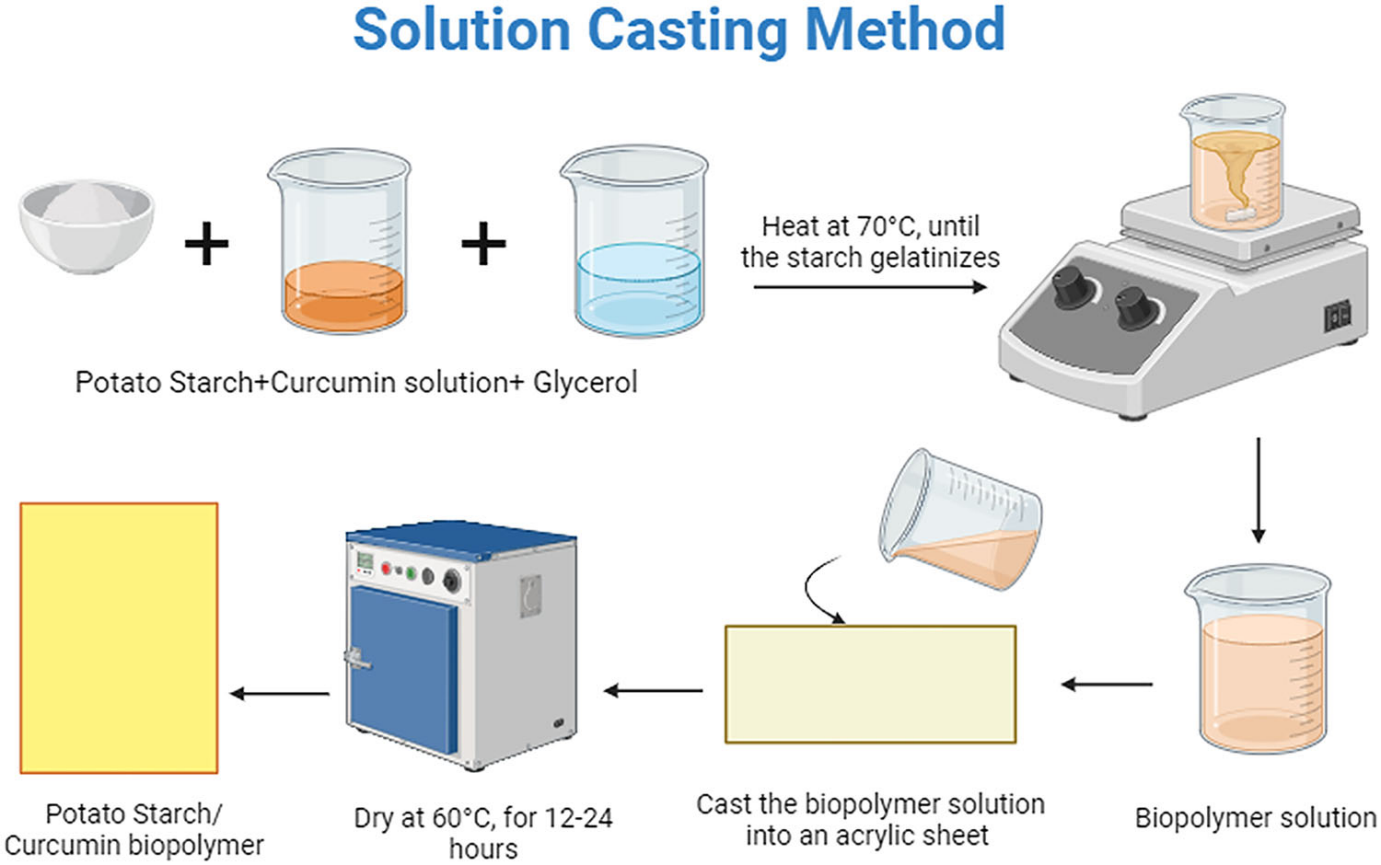
Preparation of curcumin-incorporated dressing materials via the solution-casting technique

The biopolymer dressing materials were synthesized via the solution-casting method (Fig. 1). In accordance with protocols established in previous studies [12], 8 g of potato starch was weighed and mixed with 3.5 mL of glycerol and 50 mM curcumin solution dissolved in ethanol was used to prepare the curcumin dressing material. This mixture was thoroughly mixed and brought to 100 mL with distilled water. The starch mixture was heated on a magnetic stirrer at 70 °C in the dark. The mixture was stirred continuously until the starch was evenly gelatinized. The biopolymer solution was then poured onto an acrylic casting tray and spread evenly and carefully to prevent air bubbles in the film. It was dried at 60 °C inside a hot air oven for 12–24 h and stored inside a humidity chamber until further use.

### Structural characterization

#### Thickness

The thickness of the biopolymer films was measured via a digital micrometer (0.01 m resolution; Yuzuki, Tokyo, Japan). Five different areas from a bioplastic were as taken and measured, and an average value was reported.

#### X-ray diffraction

The amorphous content and crystallinity of the starch-based biopolymer were determined via a MiniFlex benchtop XRD system (Rigaku, Japan). The 1 cm^2^ films were scanned with a 0.02° step size from 5 to 80°. X-ray diffraction was also used to study the interatomic distances and bond angles between the materials and the polymer matrix.

### Chemical characterization

#### Fourier transform infrared (FTIR) spectroscopy

An FTIR spectrometer (Bruker ALPHA FTIR spectrometer, Germany) equipped with an attenuated total reflectance (ATR) accessory was used to characterize the biopolymer. By interpreting the molecular vibrations in the polymer matrix arising from radiation at specific frequencies, (1 cm × 1 cm) films were cut and scanned to reveal the molecular bonds present. The spectra were recorded in the range of 4000–500 cm⁻¹, with a resolution of 4 cm⁻¹, and 32 scans per sample were performed using an ATR-FTIR setup. This analysis helps in determining the formation and loss of chemical bonds during the polymerization process.

### Functional characterization

#### Water solubility

The protocol was adapted from studies conducted by Chakraborty et al., and N et al. [13, 14]. 1 cm × 1 cm pieces of the biopolymer were dehydrated overnight to evaporate the water content. The initial weights of the pieces were recorded (W_i_) and consequently immersed in 30 mL of distilled water for 24 h. Excess water was wiped off by blotting paper, and the biopolymer pieces were dried for 24 h. The final weight of the biopolymer pieces was recorded (W_f_), and the water solubility of the biopolymer was calculated via Eq. (1).

1 $${\bf{Water}}\,{\bf{solubility}}({\boldsymbol{ \% }})=\frac{({{\boldsymbol{W}}}_{{\boldsymbol{i}}}-{{\boldsymbol{W}}}_{{\boldsymbol{f}}})}{({{\boldsymbol{W}}}_{{\boldsymbol{i}}})\,}* {\boldsymbol{100}}$$

#### Moisture content

In total, 1 cm × 1 cm pieces of the biopolymer were weighed to record their initial weight (W_i_). These pieces were then dried in a hot air oven at 60 °C for 24 h until a steady weight was reached. These films were weighed to record their final weight (W_f_), which was devoid of excess moisture. The moisture content of the films was calculated via Eq. (2).

2 $${\bf{Moisture}}\,{\bf{Content}}({\boldsymbol{ \% }})=\frac{({{\boldsymbol{W}}}_{{\boldsymbol{i}}}-{{\boldsymbol{W}}}_{{\boldsymbol{f}}})}{({{\boldsymbol{W}}}_{{\boldsymbol{i}}})\,}* {\boldsymbol{100}}$$

#### Tensile strength analysis

The universal testing machine records the tensile strength of the biopolymer films. The biopolymer was cut into 9 cm*2 cm pieces and analyzed via the Shimadzu Universal Texture Analyzer EZ-SX (Shimadzu Corporation, Kyoto, Japan). The results were plotted in a force (N) vs. stroke (mm) graph.

#### Water contact angle

The surface wettability and hydrophilicity of a biopolymer film are analyzed via the static water contact angle. The hydrophilicity of the film decreases as the water drops spread on the film. Using a contact angle meter (Holmarc, India), a drop of distilled water was dispensed through a syringe onto the biopolymer film and photographed after 10 s [15].

#### Biodegradation

The protocol was adapted from Chakraborty et al. [13]. Biopolymer films measuring 1 cm² were prepared and buried in pots containing garden soil within a controlled greenhouse environment. The samples were positioned approximately 2 cm beneath the soil surface to simulate natural burial conditions while ensuring uniform exposure to soil microorganisms. In a parallel setting, autoclaved soil was used as a control to assess the effect of microbial activity on degradation, with the same burial depth maintained. The physical integrity and surface morphology of the biopolymer pieces were examined at predetermined intervals, specifically on days 15 and 30, to monitor the progression of degradation over time.

### Antioxidant and radical scavenging assay

#### DPPH· (2,2-diphenyl-1-picrylhydrazyl) free radical scavenging activity

The 2,2-diphenyl-1-picrylhydrazine assay measures the free radical scavenging activity of a compound. In this assay, the ability of curcumin to bleach a purple-colored DPPH solution was measured to calculate its reduction ability. The method described by Ak and Gülçin (2008) [16] was used to determine the radical scavenging activity. The DPPH radical is absorbed at a wavelength of 517 nm, but its absorption proportionally decreases upon reduction by an antioxidant agent. To perform this assay, 0.5 mL of 0.1 mM ethanolic DPPH solution was added to 1.5 mL of ethanolic solutions of various concentrations of curcumin. These solutions were mixed thoroughly and incubated in the dark for half an hour at 25 °C. The absorbance was then measured at 517 nm against a blank sample (only DPPH· solution, without the scavenger sample). Ascorbic acid was used as a control because of its well-documented antioxidant properties. The DPPH· scavenging properties were calculated via Eq. (3).

3 $${\mathrm{DPPH}}\cdot \,{\mathrm{scavenging}}\,{\mathrm{activity}}( \% )=({\bf{1}}-\frac{{\bf{As}}}{{\boldsymbol{B}}}){\boldsymbol{* }}{\boldsymbol{100}}$$

where B is the absorbance of the blank solution (0.5 mL of DPPH solution without curcumin) and A_s_ is the absorbance of the samples containing curcumin. DPPH· proportionally decreases when exposed to radical scavengers [16].

### In vivo study

Sixteen Wistar albino rats (8–10 weeks old, male), weighing 170–200 g, were procured from the Central Animal Research Facility, Manipal Academy of Higher Education (MAHE), Manipal, India. Approval was given by the Institutional Animal Ethics Committee (**IAEC/KMC/26/2024**). The animals were kept under a 12 h light‒dark cycle under controlled conditions and provided with food and water *ad libitum*. The rats were anesthetized, and their dorsal hair was gently shaved. The shaved area was cleaned and then sterilized with an alcohol swab. An excision wound approximately 15 mm in diameter was created. The rats were then randomly grouped into 4 groups of 4 rats each:

- Group 1- Control group (with no treatment, open wound)
- Group 2- Only starch biopolymers
- Group 3 - Starch biopolymers incorporated with curcumin.
- Group 4- Positive control (human placental extract along with Neosporin spread on the starch biopolymer)

The wound, alongside the given treatment, was covered with sterile gauze and adhesive tape for proper fixation of the wound dressing. The treatment was repeated every other day to minimize animal handling and allow for better wound healing. The animals were checked for wound closure on days 0, 2, 6, 10, and 14 [10].

## Results and discussions

### Structural characterization

XRD was used to analyze the structural properties of the biopolymer. Structural characterization studies confirm whether the polymer synthesis was successful and provide insight into the polymer matrix.

#### Thickness

**Fig. 2:**
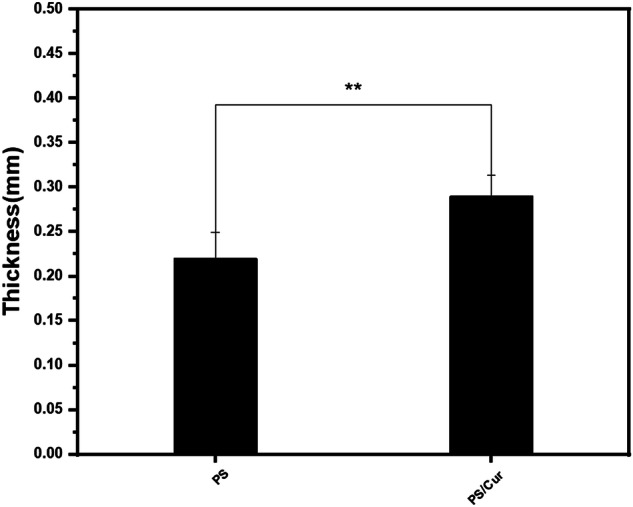
Thickness of the curcumin-incorporated biopolymer. PS- potato starch biopolymer; PS/Cur- curcumin-incorporated potato starch biopolymer. **P < 0.01

The graph (Fig. 2) illustrates the difference in thickness of the curcumin biopolymer compared to the control biopolymer, as a result of the incorporation of curcumin into the film significantly increased the thickness of the film. This was attributed to an increase in the solid mass of the polymer matrix due to the addition of curcumin to the polymer matrix.

#### XRD analysis

**Fig. 3:**
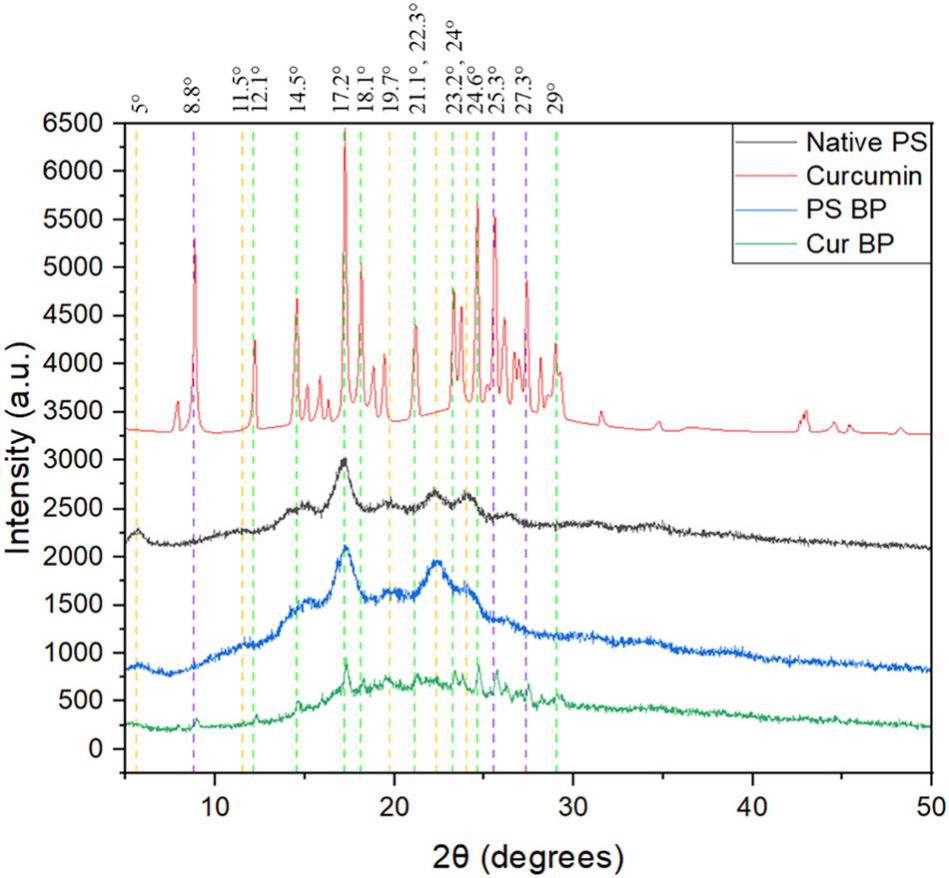
XRD of the curcumin-incorporated biopolymer

XRD analysis was performed to compare the crystallinity and amorphous content between the control potato starch film and the prepared curcumin film, native potato starch powder, and native curcumin, and the results are depicted as a diffractogram in Fig. 3. XRD analysis helps in understanding the crystalline structure of different materials. Native potato starch has a B-type crystalline structure, as evidenced by the peaks at 5°, 17.2°, 19.7°, 22.3°, and 24°. Interactions between the starch biopolymers result in the crystallinity of the biopolymer films. The crystallinity of a biopolymer dictates its flexibility and strength. Thus, the inner polymer matrix structure can greatly impact the physicochemical properties, along with the functionality of a biopolymer. This crystallinity is hindered when starch undergoes gelatinization to form biopolymer films, resulting in broad peaks, as observed via XRD. During gelatinization, the addition of additives such as glycerol and curcumin diminishes the crystallinity of the biopolymer by breaking the hydrogen bonds formed between the starch molecules and increasing chain mobility. There was a visible reduction in the peaks corresponding to 5°, 15°, 17°, 23.2°, and 24.6° upon gelatinization of potato starch. Owing to the incorporation of curcumin, the X-ray diffractogram displayed peaks at 8.8°, 12.1°, 14.5°, 17.2°, 21.1°, 27°, and 29°. The successful incorporation of curcumin into the starch matrix was indicated by the combination of starch (5°, 11.5°, 19.7°, and 24.6°) and curcumin (12.1°, 14.5°, 17.2°, 18.1°, and 29°) peaks observed in the diffractogram of the starch/curcumin biopolymer [17–19].

### Chemical characterization

#### FTIR spectroscopy

**Fig. 4:**
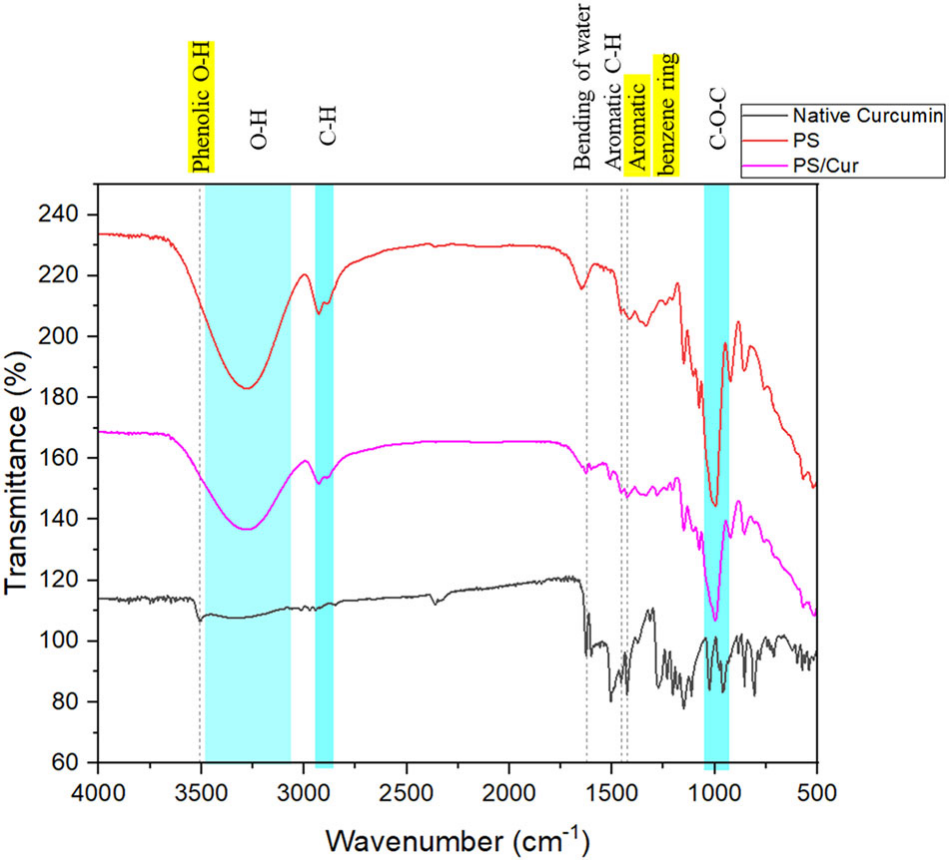
FTIR spectra of the curcumin-incorporated biopolymer

The results of FTIR spectroscopy of the curcumin-incorporated film were compared with the spectra of native curcumin and the potato starch control biopolymer film at 4000–500 cm^–1^, as shown in Fig. 4. FTIR helps in interpreting the chemical bonds present that are created and lost during the polymerization process and provides insight into the potential properties of the biopolymer. The FTIR spectrum of the potato starch control film showed peaks corresponding to O–H groups, C–H stretching vibrations, C–OC stretching, and aromatic ring stretching. The spectra of the curcumin-incorporated films were comparable, with similar peaks. There was a wide peak at approximately 3340 cm^–1^, which was attributed to the O-H group. The curcumin-incorporated film had no discernible peak at 3508 cm^–1^, even though the powdered curcumin showed a peak in that region, attributed to phenolic O-H stretching. There was a peak at 1625 cm^–1^, corresponding to aromatic C‒H bending, and peaks at 1457 and 1427 cm^–1^, both of which are linked to aromatic benzene ring stretching. Along with these peaks, the H-O-H peak for the bending of water molecules and the C-O-C peaks at 1024 cm^–1^ and 990 cm^–1^ were also observed in the transmittance spectrum. The presence of O-H and C= bonds also implies that the biopolymer can react with water, making it potentially biodegradable [12, 14, 20].

### Functional characterization

Water solubility, moisture content, water vapor transmission rate, tensile strength, and static water contact angle provide insight into the functional properties of the biopolymer film and help in testing its performance for a wide range of applications.

#### Water solubility and moisture content

**Fig. 5:**
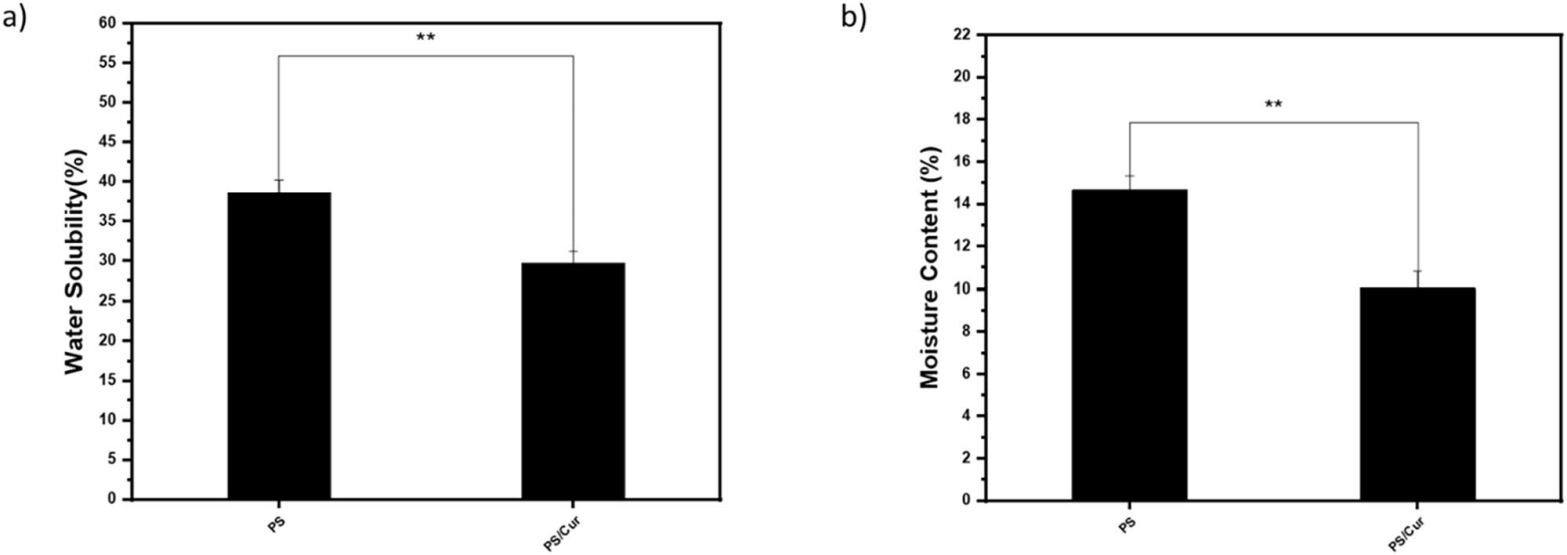
**a** Water solubility (%) and **b** moisture content (%) of the curcumin-incorporated biopolymer

The water solubility test is an important test that indicates the biodegradability of the synthesized biopolymer in water and other liquids. This property is important for bandaging applications to prevent immediate disintegration of the biopolymer upon contact with the skin and wound exudates. A water solubility (WS) test of the films was conducted by immersing the biopolymer films in distilled water for 24 h and measuring the difference in their weights. Figure 5a indicates that the incorporation of curcumin into the biopolymer film caused a significant decrease in water solubility. The incorporation of curcumin makes the film more hydrophobic, thereby lowering its ability to retain water in the matrix. The moisture content of a biopolymer film can contribute to its degradability and ability to allow for the growth of microbes on the biopolymer. A lower moisture content ensures that the biopolymer film does not disintegrate easily and prevents a moist atmosphere from allowing microbial growth. The moisture content analysis (Fig. 5b) indicated that the incorporation of curcumin caused a significant decrease in the moisture content of the biopolymer film. Curcumin forms bonds inside the polymer matrix, reducing the available polar groups to bind to water molecules [21].

#### Tensile strength

**Fig. 6:**
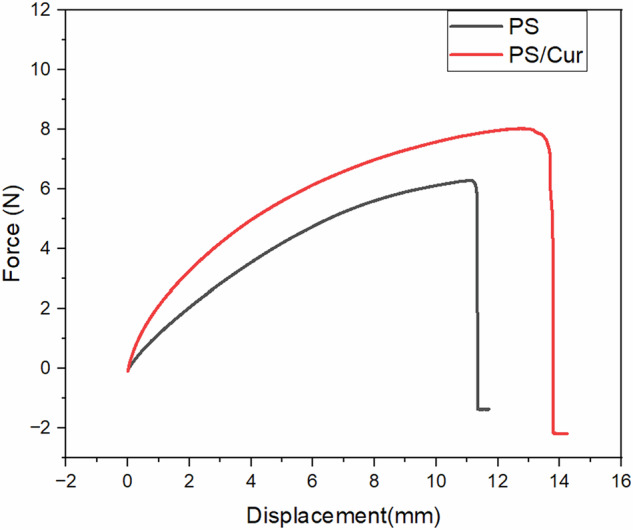
Force (N) vs. displacement (mm) graph of the curcumin-incorporated film

The tensile strength of the biopolymer was tested via a universal testing machine (UTM). The tensile strength of the film containing curcumin was tested by plotting force vs. stroke graphs via UTM analysis, as depicted in Fig. 6. Compared with the control, curcumin increased the tensile strength of the films. Curcumin acts as a cross-linking agent within the polymer matrix, strengthening the intermolecular bonds and thus increasing the tensile strength of the biopolymer.

#### Static water contact angle

**Fig. 7:**
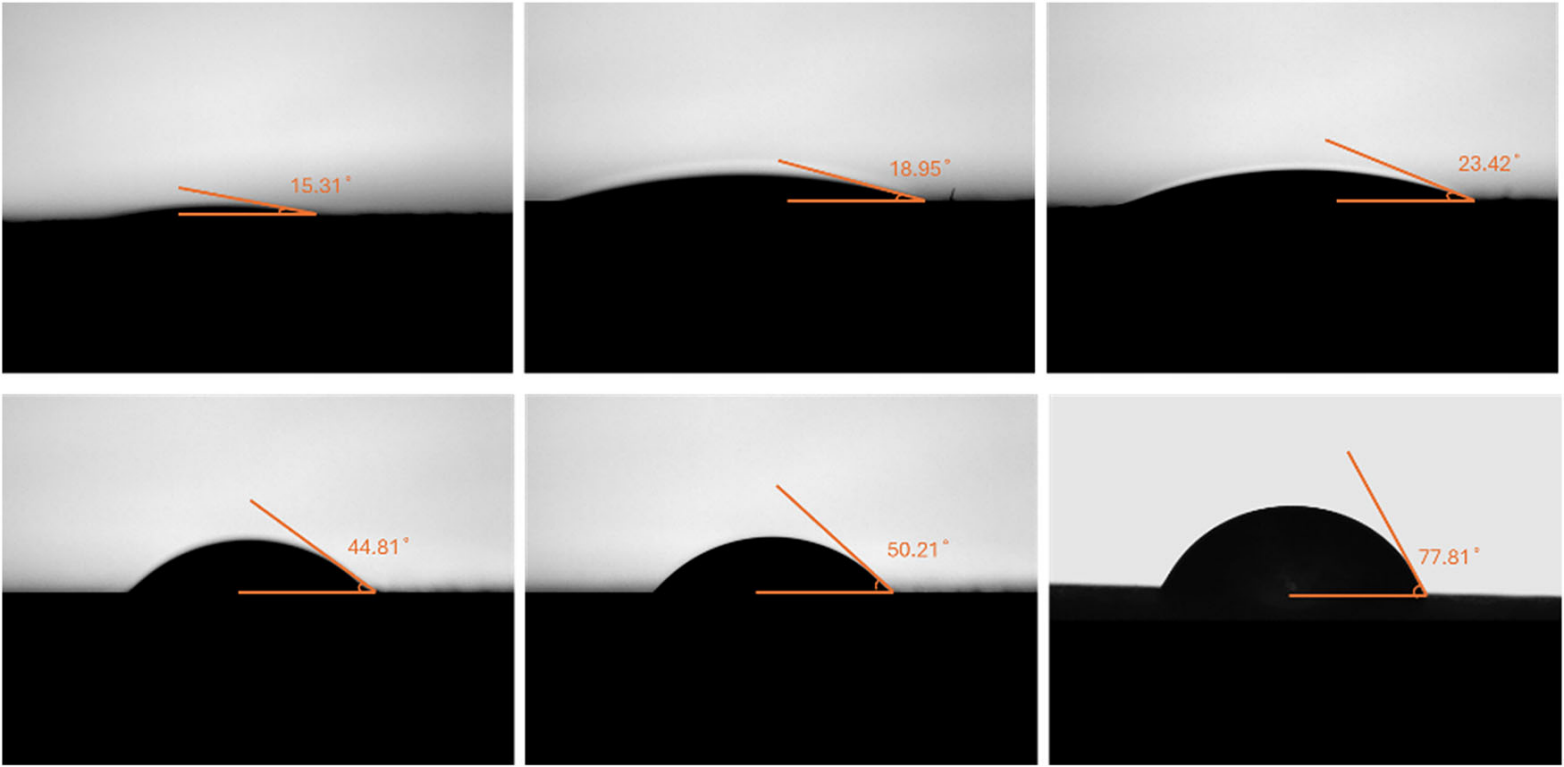
Water contact angle of the curcumin-incorporated biopolymers. (L-R- A: PS film; B: 5 μM; C: 10 μM; D: 15 μM; E: 20 μM; F: 50 mM)

The static water contact angle is a measure of the surface wettability of a polymer. This ability determines the degree of hydrophobicity of the surface and helps in determining the interactions between various particles and the polymer surface. The hydrophobicity of a material increases with increasing surface contact angle; materials having a contact angle of <90° are hydrophilic, and materials having a contact angle >90° are hydrophobic. Figure 7 shows the water contact angles of various concentrations of the curcumin-incorporated starch biopolymers. The potato starch control film, which is synthesized using only starch and glycerol, is highly hydrophilic, as evidenced by its water contact angle (15.31°). Both starch and glycerol are hydrophilic materials because of the presence of numerous hydroxyl groups in their side chains, which interact with water molecules through H-bonding. Curcumin is a hydrophobic molecule largely due to its nonpolar aromatic regions in its molecular structure. The addition of curcumin proportionally increased the water contact angle as shown in Table 1. Owing to its hydrophobic properties, curcumin makes the film more hydrophobic, increasing the water contact angle.

**Table 1:** Water contact angle of the curcumin-incorporated biopolymers

Film	Water contact angle
Potato Starch biopolymer	15.31°
Potato Starch/5 μM curcumin biopolymer	18.95°
Potato Starch/10 μM curcumin biopolymer	23.42°
Potato Starch/15 μM curcumin biopolymer	44.81°
Potato Starch/20 μM curcumin biopolymer	50.21°
Potato Starch/50 mM curcumin biopolymer	77.81°

### Biodegradation

**Fig. 8:**
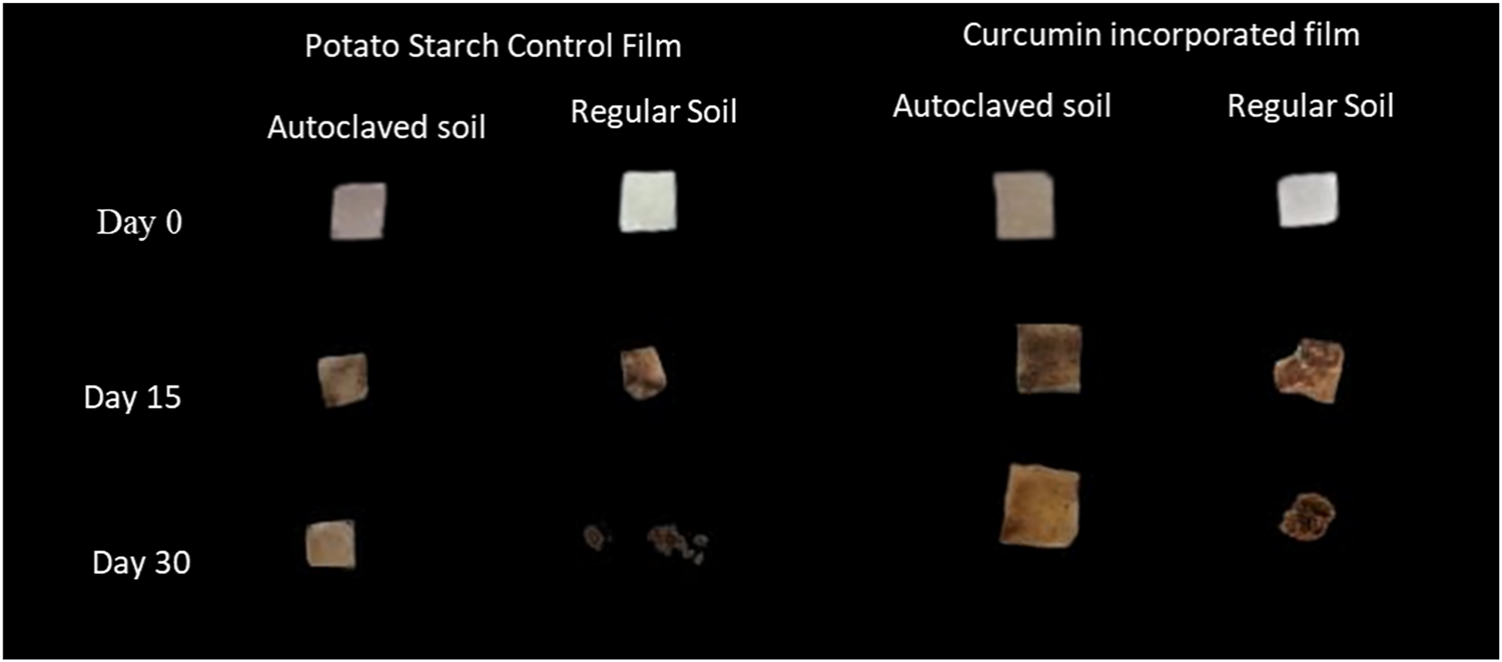
Photographs showing the biodegradation of the curcumin-incorporated biopolymer on Day 15 and Day 30

The biodegradability of the biopolymer in soil was tested by burying pieces of the biopolymer under autoclaved and normal soil. Soil burial degradation is a widely used qualitative method to evaluate the biodegradability of polymeric materials under natural environmental conditions, as it simulates the interaction with soil microorganisms and moisture. However, this method provides primarily qualitative insights, and standardized methods such as ISO 14855 or weight loss-based in vitro degradation studies are recommended for quantitative assessment of biodegradation. The pieces were observed on days 15 and 30. Curcumin slows the biodegradation process because of its hydrophobicity and antimicrobial properties. Figure 8 shows the biodegradation of the curcumin-incorporated film compared with that of the control potato starch film. The films incorporated with curcumin degrade more slowly than the control films do. The films were also compared with autoclaved soil, which showed minimum degradation on day 15 of the experiment.

### Antioxidant activity

**Fig. 9:**
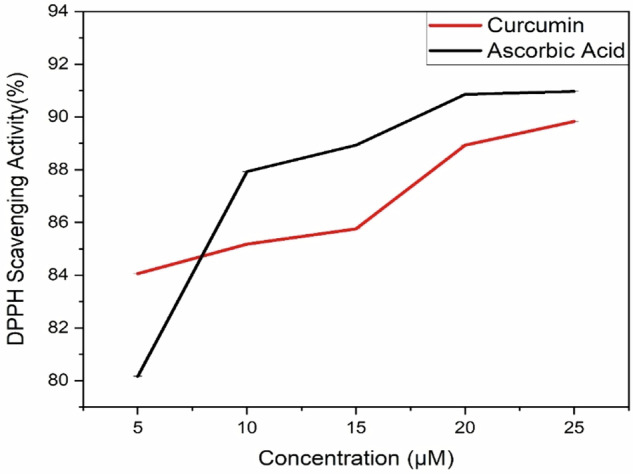
Antioxidant activity of a range of curcumin concentrations (μM) compared with that of a standard (ascorbic acid)

The antioxidant activity of curcumin was tested via a 2,2-diphenyl-1-picrylhydrazyl (DPPH) scavenging assay. The DPPH assay incorporates the use of free radicals to test the ability of a substance to act as a hydrogen donor or free-radical scavenger. The redox activity of curcumin is measured by its ability to bleach a purple-colored DPPH solution. The DPPH radical is absorbed at a wavelength of 517 nm, but its absorption proportionally decreases upon reduction by an antioxidant agent. The DPPH· scavenging activity was plotted against concentration, as shown in Fig. 9. The graphs show that curcumin is a good antioxidant, even at low concentrations (as low as 5 μM), with values comparable to those of ascorbic acid, which is used as a standard in this experiment. The curcumin plot shows a positive slope throughout, whereas that of ascorbic acid seems to reach a maximum point above 20 μM [16].

### In vivo study

**Fig. 10:**
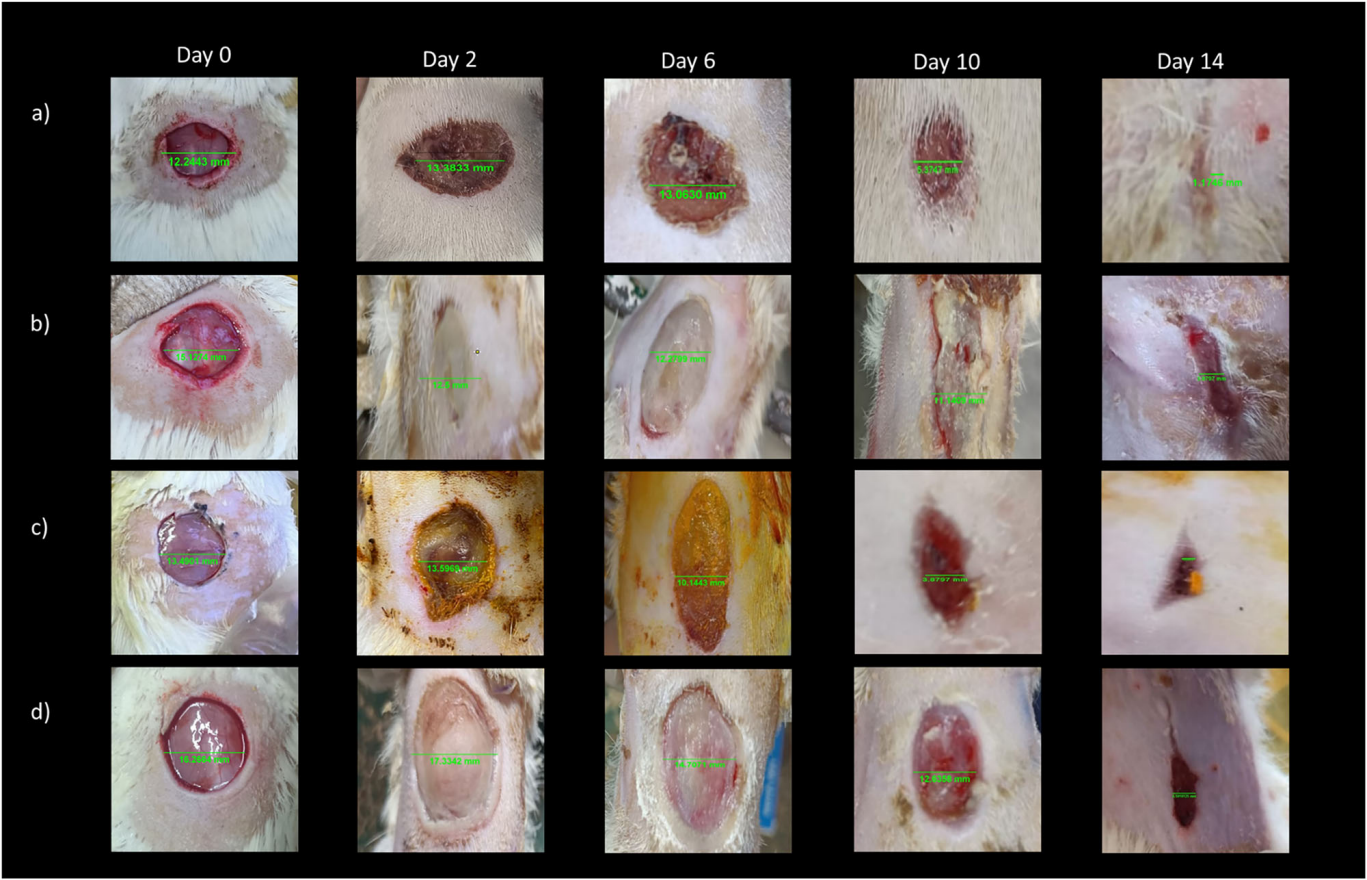
In vivo wound healing studies on Wistar rats: **a** control group, **b** only starch biopolymer-treated group, **c** curcumin-incorporated starch biopolymer-treated group, and **d** marketed pharmaceutical group

**Fig. 11:**
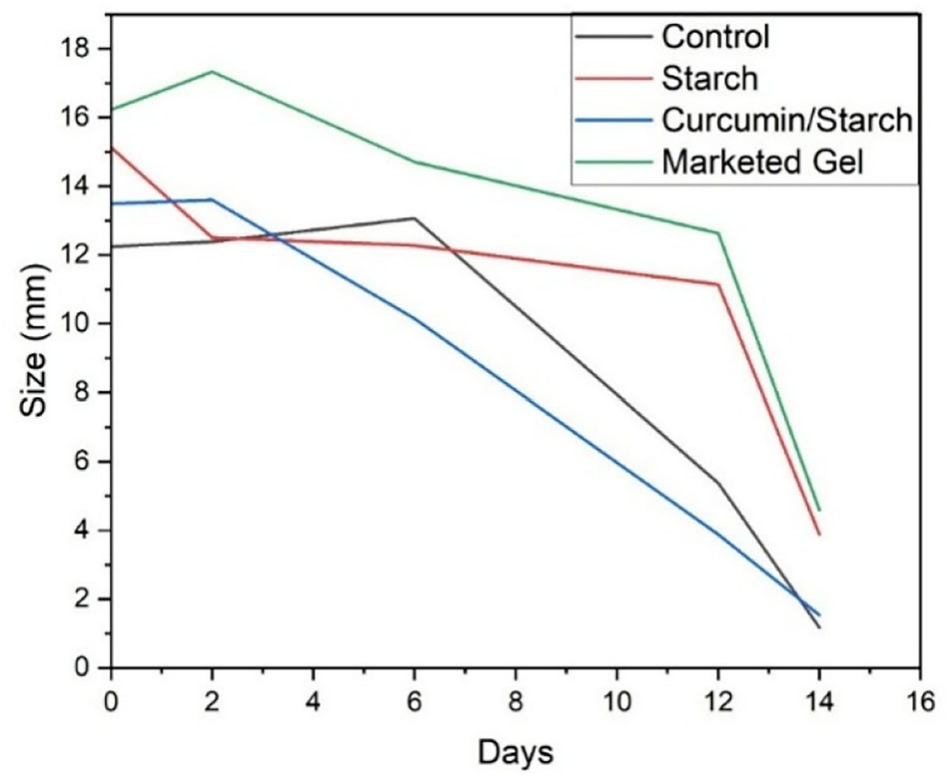
Wound closure rates of the study groups

Prior to investigating the effects of curcumin on in vivo models, curcumin was tested against an in vitro line for proof of efficacy in wound healing (Supplementary Fig. 1).The effects of the starch/curcumin biopolymer were then tested for wound closure rate in Wistar albino rats. A wound was created on the dorsal side of each rat, which was then studied over 14 days (Fig. 10). The figure shows a pictorial representation from Day 0 to Day 14 for the wound healing assay performed on Wistar albino rats. The wounds in the control group were left untreated and open without any dressing materials. There was a visible reduction in wound size, along with the formation of scabs on day 6. Compared with that on Day 0, the size of the wound on Day 10 was smaller, and the wound had healed, with a visible keloid scar on Day 14. In the starch biopolymer-treated group, mild infection was observed beginning on Day 2, causing the wound closure rate to drastically slow. In the curcumin-treated group, the wound healing rate was rapid, as shown in the size reduction chart below. The curcumin-treated group healed completely close to Day 15 and healed with a scab, under which scar-free skin formed. The curcumin-treated group also did not show any signs of infection and remained healthy throughout the study. Our study shows results similar to the work done by Dhurai et al. Tong et al. [22, 23]. The curcumin-incorporated biopolymer performed as well as the marketed pharmaceutical gel, using cost-efficient ingredients. A graph (Fig. 11) of the size of the wound (mm) vs the number of days was generated to study the wound closure rate. As shown in the graph, the wound closure rate for the starch/curcumin biopolymer was the most consistent, with a uniform decrease in size.

## Conclusion

Although many studies have investigated potato starch-based biopolymer films and the benefits of curcumin in wound healing, our research sought to combine the two to create a biodegradable curcumin-incorporated dressing material, which would accelerate wound healing and help reduce the amount of plastic waste accumulated in the biomedical waste industry. The effect of curcumin in biopolymer films increases their mechanical and functional properties while also imparting its antioxidant and wound healing properties to the film. The ease of availability of starch and curcumin also helps reduce the cost while being eco-friendly, having a very low net carbon footprint, and potentially being a source for waste management of potato peels. This study revealed that the incorporation of curcumin imparts mechanical strength, reduces the moisture content and water solubility, and slows the biodegradation of the synthesized starch biopolymer. *An* in vivo wound closure assay, which was conducted on Wistar albino rats, revealed consistent wound closure rates. This study can be upscaled for further biomedical uses if successful and has the potential to serve as a wound dressing material in conditions where wound healing ability may be impaired. The ease of availability of starch and curcumin also helps reduce the cost while being eco-friendly and having a very low net carbon footprint; thus, starch and curcumin can potentially also be sources for the waste management of potato peels. While the present study demonstrates promising wound healing efficacy of curcumin-incorporated starch biopolymers, certain limitations must be acknowledged. Histopathological analysis and detailed evaluation of inflammatory cell response were not performed in this study, which could provide deeper insights into the tissue regeneration process and underlying mechanisms. Future studies will focus on incorporating histological assessments and exudate analysis to further validate and strengthen the biological performance of the developed biomaterial.

## Supplementary information

Supplementary information

## Data Availability

The data may be available on request to corresponding author.
