# Supplementary material for: Therapeutic potential of curcumin-integrated starch biomaterials in wound regeneration
Source: J Mater Sci Mater Med. 2026 May 26;37(1):89. doi: 10.1007/s10856-026-07082-7 (PMC13388637; doi:10.1007/s10856-026-07082-7)
Supplement: Supplementary file 1 — Supplementary information [file 10856_2026_7082_MOESM1_ESM.docx]

**Therapeutic potential of Curcumin-Integrated Starch Biomaterials in Wound Regeneration**

**Tilottoma Kargupta^1,#^, Pooja N^1,#^, Shreya Shahapur^1^, Bhisham Singh Narayan^2^, Nirmal Mazumder^1,*^**

*^1^Department of Biophysics, Manipal School of Life Sciences, Manipal Academy of Higher Education, Manipal,, India*

*^2^Department of Biotechnology, Manipal School of Life Sciences, Manipal Academy of Higher Education, Manipal, India*

***#***equal contribution

**** Corresponding Email ID:*** *nirmal.mazumder@manipal.edu*

***In vitro* wound-healing assay**

**Methodology**

Wound healing assays are used to determine the effect of a drug on cell migration signals and cell-cell communications. For the wound healing assay, 1.5 x 10^5^ cells were seeded per well in a 6-well plate, and grown in complete media until they formed a uniform monolayer. The cells were then serum starved for 24 h. A scratch was created gently with the help of a sterile 200 μL tip, and the wells were washed carefully with PBS. Different concentrations of curcumin, diluted in media, were treated, and the cells were photographed for 0,6,24, 48 h (Olympus IX73 with a camera with the CellSens Programme, Hamburg, Germany) (Prasad et al., 2020).

**Results and Discussion**


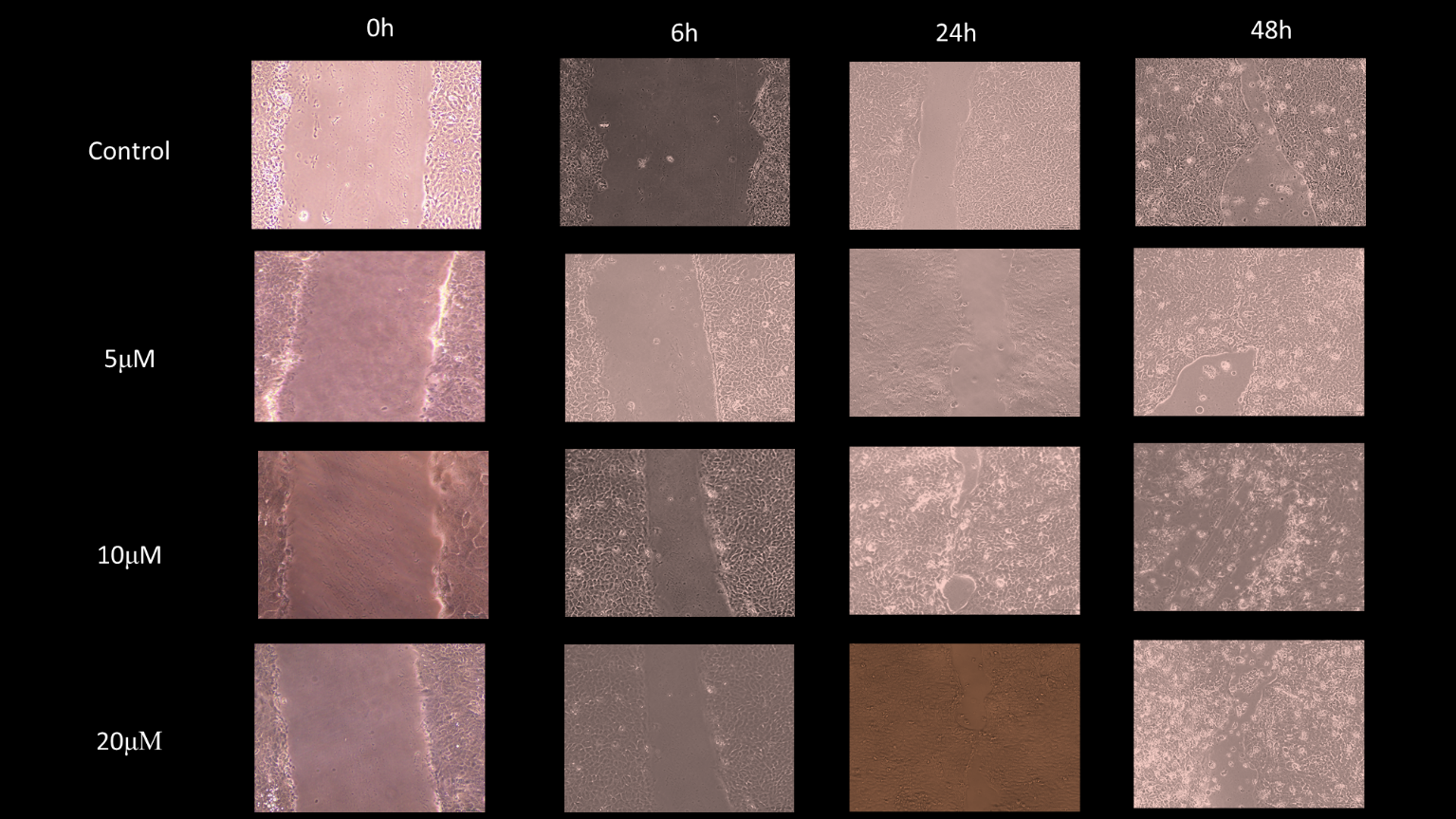


**Fig 1:** *In vitro* wound healing assay of different concentrations of curcumin

Wound healing studies can be studied *in vitro* by scratch assay. **Fig 1** represents the scratch assay was conducted using HaCaT cells, and varying concentrations of curcumin. The experiment was followed through 48 h. For all of the samples, 6 h into the assay showed no distinguishable wound closure. However, for wells treated with curcumin, irrespective of the concentration, there was a visible shortening of the wound width in 24 h. There is a positive correlation observed between the curcumin concentration and visible wound closure.

**References**

Prasad, A. S. B., Shruptha, P., Prabhu, V., Srujan, C., Nayak, U. Y., Anuradha, C. K. R., Ramachandra, L., Keerthana, P., Joshi, M. B., Murali, T. S., & Satyamoorthy, K. (2020). Pseudomonas aeruginosa virulence proteins pseudolysin and protease IV impede cutaneous wound healing. *Laboratory Investigation*, *100*(12), 1532–1550. https://doi.org/10.1038/s41374-020-00478-1
